# Post-Translational Modifications of the Mini-Chromosome Maintenance Proteins in DNA Replication

**DOI:** 10.3390/genes10050331

**Published:** 2019-04-30

**Authors:** Zheng Li, Xingzhi Xu

**Affiliations:** 1 Guangdong Key Laboratory for Genome Stability & Disease Control and Carson International Cancer Center, Shenzhen University School of Medicine, Shenzhen 518060, China; buctlz@hotmail.com; 2 College of Life Science, Capital Normal University, Beijing 100048, China

**Keywords:** mini-chromosome maintenance (MCM), post-translational modifications (PTMs), disease, cancer, DNA replication, cell cycle, genome stability

## Abstract

The eukaryotic mini-chromosome maintenance (MCM) complex, composed of MCM proteins 2–7, is the core component of the replisome that acts as the DNA replicative helicase to unwind duplex DNA and initiate DNA replication. MCM10 tightly binds the cell division control protein 45 homolog (CDC45)/MCM2–7/ DNA replication complex Go-Ichi-Ni-San (GINS) (CMG) complex that stimulates CMG helicase activity. The MCM8–MCM9 complex may have a non-essential role in activating the pre-replicative complex in the gap 1 (G1) phase by recruiting cell division cycle 6 (CDC6) to the origin recognition complex (ORC). Each MCM subunit has a distinct function achieved by differential post-translational modifications (PTMs) in both DNA replication process and response to replication stress. Such PTMs include phosphorylation, ubiquitination, small ubiquitin-like modifier (SUMO)ylation, *O*-N-acetyl-D-glucosamine (GlcNAc)ylation, and acetylation. These PTMs have an important role in controlling replication progress and genome stability. Because MCM proteins are associated with various human diseases, they are regarded as potential targets for therapeutic development. In this review, we summarize the different PTMs of the MCM proteins, their involvement in DNA replication and disease development, and the potential therapeutic implications.

## 1. Introduction

DNA replication is a complex and systematic process that ensures faithful duplication of the genome and subsequent cell division only once per cell cycle. In the first step of DNA replication, the pre-replicative complex (pre-RC) forms at the origin recognition complex (ORC), which consists of six subunits, ORC1–6; ORC1–6 then recognize and bind to autonomously replicating sequences. In early gap 1 (G1) phase, the ORC recruits the DNA replication licensing factor cell division cycle 6 (CDC6) and chromatin licensing and DNA replication factor 1 (CDT1) to the replication origins, and this recruitment subsequently promotes loading of the mini-chromosome maintenance (MCM) complex MCM2–7 onto chromatin [1]. Once loaded, CDC6 and CDT1 are released from the chromatin to prevent MCM2–7 reloading in the synthesis (S) phase and replication occurring more than once per cell cycle. In late G1 phase, cell division control protein 45 (CDC45) and the DNA replication complex Go-Ichi-Ni-San (GINS) bind to the MCM2–7 hexamer to form a CDC45/MCM2–7/GINS (CMG) complex, which activates MCM helicase activity [2]. At the same time, MCM10 brings the DNA polymerase α/primase complex to the replication origins for replication initiation [1]. During S phase, replication on leading and lagging strands relies on different DNA polymerases; the leading strand is replicated by DNA polymerase ε, while the lagging strand is replicated by DNA polymerase δ [3].

DNA replication is constantly challenged by various endogenous and exogenous stressors. Endogenous stressors include a limited source of histones and deoxyribonucleotide triphosphates (dNTPs), unusual DNA structures (G quadruplexes, hairpins, or cruciforms), and RNA–DNA hybrids (R loops) [4]. Exogenous stressors include drugs or inhibitors that target DNA polymerase (such as aphidicolin, clofarabine) or nucleotide synthesis (such as hydroxyurea); however, most of these agents are widely used in cancer therapy. Replication stress can cause replication fork stalling and even collapse if the stress cannot be resolved. During fork stalling, the nearby dormant MCM2–7 complex becomes activated to ensure replication. Fork stalling also activates the DNA replication checkpoint, which mostly depends on the ataxia telangiectasia and Rad3-related (ATR)–checkpoint kinase-1(CHK1) pathway. Here, the ATR–CHK1 pathway has two functions; it regulates dNTP synthesis to ensure sufficient supply for replication [5], and protects the fork from aberrant remodeling, fork cleavage, and single-strand DNA (ssDNA) formation [4]. The collapsed replication fork generates DNA double-strand breaks (DSBs), which activate the DNA damage response mediated by the phosphatidylinositol 3-kinase (PI3K)-like protein kinase family, including ataxia telangiectasia mutated (ATM), ATR, and the DNA-dependent protein kinase (DNA-PK) to modulate cyclin-dependent kinase (CDK) activity and post-translational modifications (PTMs) of the pre-RC for maintaining faithful replication [6,7].

Various mutations, deletions, and amplifications of the genes encoding replication factors, particularly the MCM complex, are associated with human diseases, such as cancer; these factors may, thus, serve as potential targets for the development of therapeutic inhibitors [8]. Here, we summarize the PTMs of the MCM family members, their involvement in DNA replication and associated human diseases, and the potential therapeutic implications.

## 2. Overview of the MCM Proteins

MCM proteins were first found in yeast as a protein family consisting of 10 members (MCM1–10) [9]. MCM1 ortholog is a serum response factor in mammalian cells that acts as a transcription factor [10]. The MCM2–7 complex is the core component of the CMG helicase complex, which is needed to unwind DNA. The six related MCM proteins are loaded onto DNA in G1 phase in a double, ring-shaped heterohexamer head–head configuration, with an anti-clockwise order of MCM2–MCM6–MCM4–MCM7–MCM3–MCM5. The complex is inactive (dormant) in G1 phase and activated in the G1/S transition through multiple PTMs, such as Dbf4-dependent kinase (DDK) and CDK-dependent phosphorylation, and further extension of the CMG complex to include CDC45 and GINS [2,11,12,13]. The majority of the MCM2–7 hexamer (~90%) remains dormant on the chromatin and ready for local activation when the replication fork is stalled or slowed; this feature is important for maintaining genome stability and integrity during DNA damage and replication stress [14,15,16]. 

The role of the MCM8–9 complex in DNA replication is still controversial. MCM8 depletion in human cells compromises chromatin loading of CDC6 and MCM2–7, suggesting that MCM8 is important for pre-RC assembly [17]. Although both MCM8 and MCM9 in *Xenopus* egg extracts bind with chromatin in late S phase, depletion of both has no impact on MCM2–7 complex loading, but reduces the chromatin-bound CDC45 and GINS2 levels [18]. The simplest explanation for these inconsistent findings could be due to different species used in the experimental assays. In addition, the MCM8–9 complex is involved in homologous recombination-mediated double-strand break (DSB) repair [19,20] and DNA inter-strand cross-linking [20]. During replication, MCM10 tightly binds the CMG complex and is required for CMG helicase activity [21,22]. Recently, Mayle et al. found that MCM10 has annealing activity and is able to block fork regression [23,24].

In addition to replication, MCM proteins may also participate in the DNA damage response [23,25]. MCM2 and MCM3 are direct ATM/ATR substrates, and loss of MCM10 causes accumulation of DNA damage during replication [23]. Moreover, MCMs directly interact with cellular tumor antigen p53 (TP53)-binding protein 1 (53BP1) and Rad51, and depletion of MCMs leads to reduced 53BP1 and Rad51 foci formation upon DNA damage [25,26]. These data support the important role of MCM proteins both in DNA replication and the DNA damage response.

## 3. Involvement of the MCM Proteins in Human Disease

Ensuring high-fidelity replication that occurs once per cell cycle and a proper response to spontaneous or external replication stress is essential to maintain genome integrity and normal cell growth and proliferation. Should these processes fail, various human diseases will ensue. Numerous genetic alterations of the MCM complex were uncovered by genome sequencing (Table 1). Gao et al. identified a heterozygous missense mutation (Arg44 to Cys) in the MCM2 gene that specifically segregated with eight affected members of a four-generation Chinese family with autosomal dominant non-syndromic deafness (DFNA70) [27]. DFNA70 is characterized by non-syndromic sensorineural and post-lingual progressive hearing loss caused by damage to structures in the inner ear, with no additional effects on other tissues [28]. Overexpression of the MCM2 (Arg44 to Cys) mutant induces apoptosis but has no obvious impact on cell proliferation, which leads to the hypothesis that MCM2 mutation-induced apoptosis gives rise to progressive hearing loss [27].

Casey et al. and Hughes et al. independently identified a rare pathogenic variant, MCM4 (c.71-1insG), which segregated with patients with natural killer cell and glucocorticoid deficiency with DNA repair defect (NKGCD) [29,30]. NKGCD is an autosomal recessive disorder characterized by microcephaly, decreased numbers of natural killer cells, and recurrent viral infections [31]. The MCM4 insertion generates a stop codon in the N-terminus, resulting in a truncated protein that may lead to DNA repair disorder and cell-cycle delay, but without compromising MCM2–7 complex formation [29,30,31,32]. In a mouse model, it was found that a Chaos3 (chromosome aberrations occurring spontaneously 3) mutation in MCM4 (Phe345 to Ile) resulted in genome instability and mammary adenocarcinomas [33]. 

MCM5 is involved in Meier–Gorlin syndrome-8 (MGORS8)—a form of primordial dwarfism (PD) in which growth problems begin before birth [34]. Vetro et al. identified biallelic variants in MCM5 (c.850_851delAG in exon 7; c.1397C–T transition in exon 11) in a 4.75-year-old boy with MGORS8 [35]. Although there are no reports on the direct relationship between MCM5 variants and human disease, decreased MCM5 expression correlates with a growth defect in zebrafish [36] and c.850_851delAG mutant embryos show cell-cycle progression defects. In yeast, MCM5 depletion is lethal, and re-introduction of a p.Thr501 to Ile mutant (corresponding to the Thr466 to Ile variant in human) is not sufficient as a rescue. These phenomena lead to the hypothesis that MCM5 is involved in Meier–Gorlin syndrome via a DNA replication defect [34].

MCM8 and MCM9 are both implicated in premature ovarian failure (POF) that is characterized by loss of menstrual function before 40 years of age. Three mutations were identified in MCM8 (Pro149 to Arg; c.1954-1G–A in intron 14; c.1469-1470insTA) and three mutations in MCM9 (c.1732þ2T > C; Arg132 to Ter; Glu495 to Ter) that segregate with this disease [37,38,39,40,41]. Furthermore, Al Asiri et al. found that fibroblasts derived from a patient with POF carried more chromosomal breaks than fibroblasts derived from the patient’s unaffected daughter [40]. A Pro149 to Arg mutation in MCM8 compromises its recruitment to DSB sites. It was also found that both *Mcm8^−/−^* and *Mcm9^−/−^* mice exhibit impaired homologous recombination (HR)-mediated DNA repair, leading to defects in gametogenesis [42]. These works reveal the important functions of MCM8 and MCM9 in ovary maturation. Thus, it is considered that dysfunctional mutations in MCM8 and MCM9 can lead to genomic instability and POF disorder potentially due to a crucial function of the MCM8–MCM9 complex in HR repair [40]. 

Although these mutations in the MCM genes are associated with human diseases (Table 1), the underlying molecular mechanisms regarding how they contribute to the initiation and development of these diseases are still unclear. Urgent work is required to understand how these genes contribute to disease in order to develop effective therapeutic strategies.

## 4. Involvement of the MCM Proteins in Human Cancer

Multiple studies revealed that dysfunctional alterations to the MCM genes may have a notable impact on tumorigenesis in various cancers [43,44,45]. A recent review highlighted that CMG complex-related genes are highly overexpressed in various cancers [8]. We explored The Cancer Genome Atlas to summarize the alterations (mutations, amplifications, and deletions) in MCM2–10 genes in different cancers (Table 2). In 10 cancer cohorts including >250 patients, we found that at least one of the MCM genes was amplified in head and neck squamous cell carcinoma, esophageal adenocarcinoma, hepatocellular carcinoma, invasive breast carcinoma, and pancreatic adenocarcinoma. In cutaneous melanoma, uterine endometrioid carcinoma, and mucinous carcinoma, we found that the MCM subunits were mutated at a high frequency. This finding was especially the case in prostate adenocarcinoma, where we found a high frequency of MCM6 (3.61%) and MCM9 (6.16%) deletions in 1803 cases. We also detected a high percentage of amplifications in MCM4 and MCM7 genes in various cancers, such as serous ovarian cancer and head and neck squamous cell carcinoma, implying that MCM7 and MCM4 may have a role in promoting tumorigenesis and could serve as credible prognostic markers for cancer diagnosis. These datasets support that alterations to MCM genes are strongly associated with tumorigenesis. We believe that studies that will provide the detailed molecular mechanisms of MCM alterations in cancer are needed to develop better cancer therapies.

Replicative helicases and polymerases have important roles in normal cell proliferation; thus, their abnormal expression or dysfunctional alterations may promote tumorigenesis. Many studies are focusing on searching for therapeutic targets and developing small molecules for cancer treatment, and MCM proteins are of great interest as potential targets. Seo et al. described nine small molecules—iovastatin, metformin, genistein, trichostatin A, bromodomain and extraterminal domain proteins inhibitor (BETi), breviscapine, heliquinomycin, ciprofloxacin, and widdrol—to target the MCMs via three main strategies [8]. The first strategy focuses on downregulating MCM expression to suppress tumor cell growth. The second strategy aims to inhibit MCM enzymatic activity [46]. The third strategy is based on developing a cancer vaccine. Of these nine molecules, heliquinomycin and ciprofloxacin inhibit MCM4/6/7 helicase activity [47,48,49], while the others negatively regulate the MCM2–7 complex or subunit (MCM2, MCM5, MCM7) expression [50,51,52,53,54,55]. Although ciprofloxacin, a fluoroquinolone inhibitor, was proven to be the best molecular scaffold to modulate MCM2–7 helicase activity, other analogs still need to be developed to achieve optimal MCM2–7 inhibition [56]. Meanwhile, the MCM PTM sites that are involved in promoting replication are likely potential targets for inhibitors or peptides and could ultimately have effective therapeutic implications in the context of human cancers.

## 5. MCM Protein PTMs Modulate DNA Replication and the Replication Stress Response

### 5.1. MCM Phosphorylation

**Figure 1:**
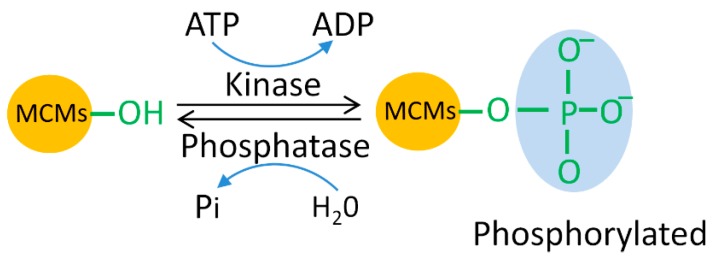
Mini-chromosome maintenance (MCM) phosphorylation. ADP, adenosine diphosphate; ATP, adenosine triphosphate.

Phosphorylation is the best studied PTM to occur on MCM proteins in human cells. Phosphorylation occurs throughout the cell cycle, especially in G1 and S phase when replication is initiated and progressed (Figure 1). MCM phosphorylation is mainly mediated by CDKs and cell division cycle 7-related protein kinase (CDC7) and regulated by the ATM/ATR checkpoint pathways (Table 3).

#### 5.1.1. CDK-Dependent Phosphorylation of MCMs

CDKs are serine/threonine kinases that bind a specific cyclin to regulate different phases of the cell cycle; they phosphorylate multiple substrates to precisely control cell-cycle progression [57]. MCM2 Ser-139 and MCM3 Ser-711 are sites phosphorylated by CDK2 [58]. Lin et al. found CDK1 phosphorylated MCM3 at Ser-112, Ser-611, and Thr-719 [59]. Phosphorylation on Ser-112 promotes MCM2–7 complex formation and subsequent chromatin loading. Li et al. found that cyclin B/CDK1 phosphorylated MCM3 at Thr-722 to promote MCM3 chromatin loading [60]. Schumann et al. found that CDK1-mediated MCM3 phosphorylation at Ser-112 and Thr-722 is essential for peptidyl-prolyl *cis–trans* isomerase never in mitosis A (NIMA)-interacting 1 (Pin1) binding [61]. Pin1 is required for MCM3 chromatin loading in early S phase, and Pin1 depletion inhibits MCM3 disassembly from chromatin in gap 2 (G2)/mitosis (M) phase. These data reveal that a phosphorylation-dependent interaction between Pin1 and MCM3 regulates MCM3 chromatin binding and disassembly in a cell-cycle-dependent manner.

MCM7 phosphorylation at Ser-121, which is mediated by cyclin E/CDK2, also promotes its chromatin loading and proper mitotic exit in M phase [62]. Excessive levels of MCM3 or MCM7 can block S-phase entry and activate the checkpoint pathway [60,62]. MCM7 is also strongly phosphorylated at Ser-365 by CDK2, as shown in vivo [63], but the functional consequences of this phosphorylation on DNA replication are illusive.

Komamura-Kohno et al. reported that CDK1 phosphorylates MCM4 at Thr-7, Thr-19, Ser-32, Ser-88, and Thr-110 (predominantly during G2/M phase), while CDK2 phosphorylates MCM4 at Ser-3 and Ser-32 (enriched in interphase) [64]. However, MCM4 pS3 and pS32 do not associate with DNA replication because the phosphorylation signal does not colocalize with replicating DNA [64]. A study by Moritani and Ishimi, however, did find that MCM4 phosphorylation at Thr-7, Thr-19, Ser-32, and Thr-110 is important for releasing the MCM complex from chromatin [65]. This finding reveals that site-specific MCM4 phosphorylation has distinct roles during cell-cycle progression.

#### 5.1.2. CDC7-Dependent Phosphorylation of MCMs 

CDC7 phosphorylates many critical substrates that regulate the G1/S phase transition and DNA replication [66,67]. Cdc7–Dbf4 (Protein Dbf4 homolog in human, activator of S-phase kinase) phosphorylates MCM2 at Ser-4, Ser-5, and Ser-7 and promotes MCM2 chromatin loading during cell-cycle reentry [63]. These phosphorylation events occur prior to CDC7-dependent phosphorylation on Ser-27, Ser-41, and Ser-139. Overexpression of phosphorylation-defective mutants MCM2 (S27A), MCM2 (S41A), and MCM2 (S139A) failed to promote DNA replication; however, they did not impact on MCM2 chromatin loading [63,68]. Montagnoli et al. and Charych et al. found that CDC7 phosphorylates MCM2 at Ser-40, Ser-53, and Ser-108, both in vivo and in vitro [69,70]. CDC7 depletion drives a slight change in MCM2 phosphorylation levels at Ser-108; as Ser-108 is a target of ATR, these data suggest that Cdc7 and ATR coordinately regulate replication events [71]. Cho et al. found that Cdc7 phosphorylates MCM2 at two major sites (Ser-5, Ser-53) and three minor phosphorylation sites (Ser-4, Ser-7, Thr-59) in vitro [72]. They also identified an additional CDC7-dependent phosphorylation at Ser-26 generated by cyclin E/CDK2-dependent phosphorylation at Ser-27. This sequential action of cyclin-dependent and CDC7 kinases may precisely control the initiation of DNA replication.

#### 5.1.3. ATM/ATR-Dependent Phosphorylation of MCMs

Many studies showed that the MCM complex is involved in the ATM/ATR signaling pathways [7,71]. Cortez et al. found that MCM3 is phosphorylated by ATM at Ser-535 upon irradiation, while MCM2 phosphorylation at Ser-108 is partially mediated by ATR upon ultraviolet (UV) light exposure or hydroxyurea treatment [71]. Further, Matsuoka et al. screened for irradiation-induced phosphorylation at potential serine/threonine-glutarnine (S/TQ) sites within MCM3 (Ser-681, Ser-728, Ser-734), MCM6 (Thr-791), and MCM7 (Ser-549) [73]. These data suggest that ATM/ATR-dependent phosphorylation of the MCM complex may serve as a platform to connect the checkpoint kinases to DNA replication.

Han et al. found that MCM3 is phosphorylated by CHK1 at Ser-205, which negatively regulates DNA replication [74]. An S205A mutant overexpressed in MCM3-depleted cells increased replication protein a foci formation compared to wild-type cells upon exposure to the DNA polymerase inhibitor, aphidicolin [74]. This finding indicates that MCM3 phosphorylation at Ser-205 also has a negative role in checkpoint signaling. Finally, Blasius et al. identified that MCM5 Thr-633 is a potential phosphorylation site for CHK1, but the impact of this phosphorylation mark awaits further investigation [75].

#### 5.1.4. Other Kinase-Dependent Phosphorylation of MCMs

Casein kinase II (CK2) is a ubiquitous serine/threonine kinase that participates in multiple pathways, such as apoptosis and cell survival [76]. Montagnoli et al. found that casein kinase II subunit α (CSNK2A1) specifically phosphorylates MCM2 at Ser-139 [69]. Because hydroxyurea and etoposide treatments have no impact on Ser-139 phosphorylation, Ser139 may not regulate checkpoint signaling. Death-associated protein kinase (DAPK) is a serine/threonine kinase involved in caspase-dependent cell death, cell adhesion, and migration. DAPK1 phosphorylates MCM3 at Ser-160 but has no impact on MCM3 chromatin loading [74,77]. Drissi et al. identified 14 etoposide-induced phosphorylation sites on MCM proteins: MCM2 (Ser-26, Ser-27, Ser-40, Ser-41, Ser-108, Ser-139, Ser-170); MCM3 (Ser-672, Ser711, Thr-713, Ser-728); MCM4 (Ser-3); MCM6 (Ser-13, Ser-762) [78]. The function of these phosphorylation marks in the DNA damage response and replication requires further investigation.

### 5.2. MCM Ubiquitination

**Figure 2:**
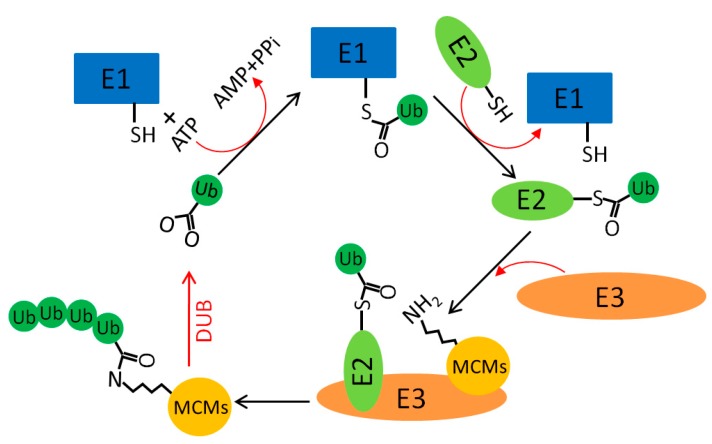
MCM ubiquitination.

The ubiquitination system is involved in various cellular processes, such as protein stability, cell-cycle progression, DNA replication, and cellular metabolism [100,101,102]. Ubiquitin is a small peptide (8.6 kDa) that can covalently conjugate with a lysine residue of a substrate protein through three enzymatic steps: activation by ubiquitin-activating enzymes (E1s), conjugation by ubiquitin-conjugating enzymes (E2s), and ligation by ubiquitin ligases (E3s). Ubiquitination can be monomeric or polymeric (Figure 2) [103]. Linkage type through different lysine residues in ubiquitin (Lys-6, Lys-11, Lys-27, Lys-29, Lys-33, Lys-48, Lys-63) and the first methionine defines different poly-ubiquitination chains and function. All the human MCM proteins are ubiquitylated in human cells [104,105,106], and the ubiquitination status of some MCM proteins changes upon DNA damage and replication stress [78] (Table 4). This finding suggests that MCM ubiquitination may have an important role in regulating replication progression and genome stability.

Wagner et al. identified several ubiquitination sites in MCM proteins involved in proteasomal degradation [106]. Mulvaney et al. identified MCM3 as a substrate for KEAP1–CUL3–RBX1 (KEAP1: Kelch-like Erythroid-derived Cap-N-Collar homology protein (ECH)-associated protein 1; CUL3: Cullin-3; RBX1: E3 ubiquitin protein ligase) complex-mediated ubiquitination both in vivo and in vitro [107]. KEAP1 binds to MCM3 throughout the cell cycle and serves as an adaptor protein to link MCM3 to the CUL3-based E3 ligase. The researchers identified several sites (Lys-229, Lys-270, Lys-283, Lys-351, Lys-435, Lys-748) that are responsive to KEAP1-dependent ubiquitination. Unexpectedly, it was determined that KEAP1-mediated ubiquitination confers little impact on total MCM3 protein stability or subcellular localization [107]. Additional specific ubiquitination sites of MCM3 (Lys-315, Lys-655, Lys-656) were found to increase upon etoposide treatment, indicating a link between MCM3 and the DNA damage response [78].

MCM7 ubiquitination mostly serves to ensure replication termination and replicative helicase disassembly [108,109,110]; any replication stress will block this termination-specific polyubiquitylation [108]. In budding yeast, SCFDia2 (Skp1/Cullin/F-box protein) ubiquitinates MCM7 in vivo and in vitro to promote CMG disassembly [109,111]. Although MCM7 polyubiquitination mostly occurs on lysine 48 (K48) of ubiquitin, it does not cause MCM7 proteolysis [108]. K48-linked poly-ubiquitinated MCM7 is recognized by the p97 complex; then, the CMG complex and other replisome components are removed from chromatin [108,110,112,113]. These events terminate DNA replication. Recently, Wu et al. identified a E3 ubiquitin protein ligase: tumor necrosis factor receptor-associated factor-interacting protein (TRAIP) in *Xenopus* egg extracts, which ubiquitinates MCM7 when the replication fork is stalled at the inter-strand crosslink (ICL) sites [114]. This TRAIP-mediated MCM7 ubiquitination leads to two different ICL-repair pathways; the short ubiquitin chains trigger direct cleavage by recruiting endonuclease 8-like protein 3 (NEIL3) glycosylase, while the long ubiquitin chains are recognized by the p97 complex, and the crosslinks undergo Fanconi anemia repair pathway [114]. 

MCM10 ubiquitination is induced by replication stress. In human cells, UV irradiation triggers MCM10 proteolysis, which ultimately inactivates the replication machinery [115,116]. This downregulation of MCM10 occurs via ubiquitination mediated by the Cul4–Roc1–DDB1 (Cul4: Cullin-4; Roc 1: E3 ubiquitin protein ligase; DDB1: DNA damage-binding protein 1) complex. UV-induced MCM10 degradation can be rescued by exposure to an ATM/ATR inhibitor and UCN01 (CHK1 inhibitor), suggesting that this downregulation may be caused by ATR and CHK1 kinases [115,117].

Although many studies showed that MCM ubiquitination is involved in regulating replication progression and genome stability, research on MCM ubiquitination in human cells is scarce. More research is now needed into the biological function of MCM ubiquitination on DNA replication and genome stability.

### 5.3. MCM UFMylation

**Figure 3:**
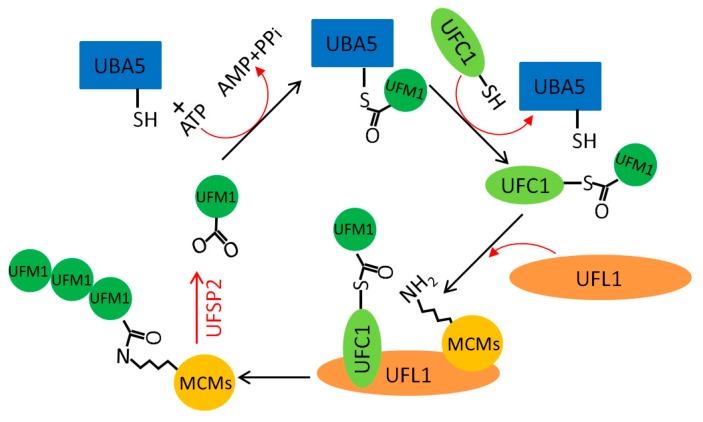
MCM modification by the ubiquitin-fold modifier 1 (UFMylation).

Modification by the ubiquitin-fold modifier 1 (UFM1), known as UFMylation, is a recently identified ubiquitin-like (UBL) PTM [118]. UFMylation utilizes the ubiquitin-like modifier-activating enzyme (UBA5), the UFM1-conjugating enzyme 1 (UFC1), and the UFM1-specific ligase 1 (UFL1) as the only E1–E2–E3 enzymatic UFMylation cascade in human cells, while the UFM1-specific protease 2 (UfSP2) serves for deUFMylation (Figure 3) [119,120]. UFMylation has important regulatory roles in endoplasmic reticulum homeostasis [121,122], hematopoiesis [123], vesicle trafficking [124], liver development [125], G-protein-coupled receptor signaling [126], transcription [127,128], mitosis [129], and autophagy [130]. We and others recently reported that UFMylation of MRE11 at K282 or histone H4 at K31 promotes ATM activation in response to DSBs [131,132]. These works uncovered for the first time that UFMylation functions at the very early stage of the DNA damage response. 

Global screening of UFMylation substrates identified MCM3 and MCM7 as potential substrates [129,133]. Our lab is actively involved in investigating MCM UFMylation and its potential roles in DNA replication under unperturbed conditions and in the replication stress response.

### 5.4. MCM Sumoylation

**Figure 4:**
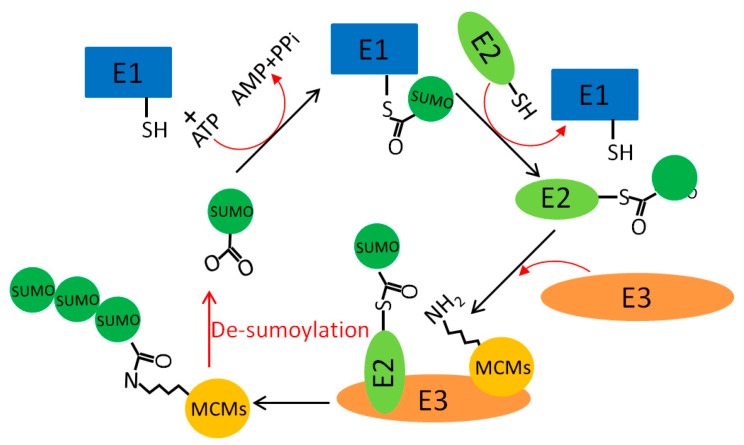
MCM modification by small ubiquitin-like modifier (SUMOylation).

The small ubiquitin-like modifier (SUMO) protein belongs to the ubiquitin-like protein family. SUMOs are covalently attached to or detached from substrates, regulating various cellular processes [134]. In human cells, SUMOs have four isoforms: SUMO-1, SUMO-2, SUMO-3, and SUMO-4. The SUMO precursor undergoes cleavage by the Sentrin-specific protease (SENP) to remove extra C-terminal amino acids before it becomes mature. Like ubiquitination, sumoylation needs three enzymatic steps (E1 activation, E2 conjugation, E3 ligation) to covalently add SUMO moieties onto a substrate (Figure 4) [135]. MCM sumoylation is highly conserved across different species [136,137,138]. 

In *Saccharomyces cerevisiae*, MCM2 and MCM3 are sumoylated by the SUMO ligase Mms21, and MCM6 is sumoylated by the SUMO ligases Siz1 and Siz2 [139]. Decreased MCM sumoylation enhances the accumulation of gross chromosome rearrangements [139]. Wei and Zhao found that only the chromatin-bound MCM subunits are sumoylated [140]. Here, MCM sumoylation is enriched in G1 phase and decreased at the entry to S phase when MCM phosphorylation is increased, suggesting that sumoylation may have an inhibitory role to prevent replication initiation in G1 phase. Reduced sumoylation activity during S phase depends on DDK and the GINS subunit, Psf2. Conversely, increased MCM sumoylation promotes protein phosphatase 1 (PP1) recruitment to the MCM complex, which leads to decreased CMG protein levels and subsequently inhibited DNA replication initiation [140]. MCM sumoylation is also responsible for DNA damage signaling and the cellular response to cytotoxic stress [136,138]. In yeast, upon DNA damage induced by the DNA methylation agent methyl methanesulfonate, MCM4 and MCM5 monosumoylation and MCM2 and MCM6 polysumoylation levels significantly increase [136]. In human cells, sumoylated MCM2, MCM3, MCM4, and MCM7 are responsive to a heat shock, indicating that MCM sumoylation may have a role in the cellular response to cytotoxic stress [138].

Together, these findings reveal that sumoylation may have an important safeguarding role to maintain genome stability and tolerate cytotoxic stress by inactivating helicase to negatively regulate DNA replication. As most of the MCM sumoylation sites (Table 5) are also potential ubiquitination sites, this complicated relationship may induce a different MCM “status” that helps to precisely control replication progression.

### 5.5. MCM O-N-acetyl-D-glucosamine (GlcNAc)ylation

**Figure 5:**
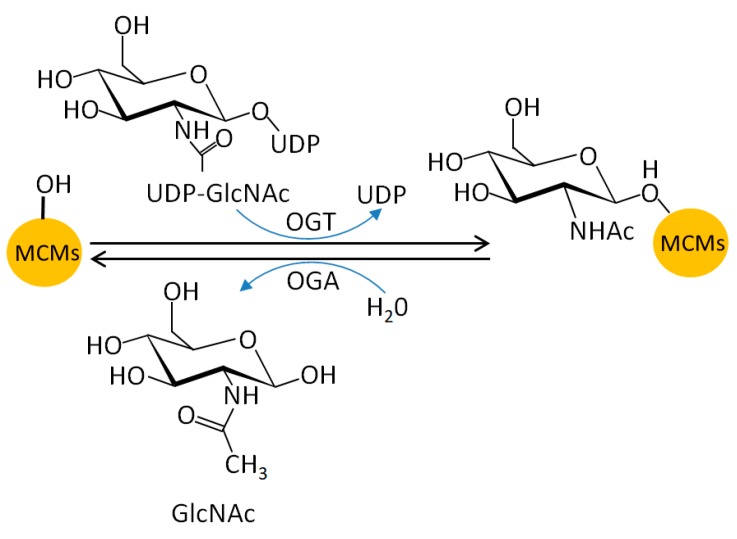
MCM *O*-N-acetyl-D-glucosamine (GlcNAc)ylation. OGT, *O*-GlcNAc transferase; OGA, *O*-GlcNAcase; UDP, uridine diphosphate.

*O*-GlcNAcylation (*O*-linked β-*N*-acetylglucosaminylation) is catalyzed by *O*-GlcNAc transferase (OGT) (Figure 5) and is a rapid responsive modification to nutrient deprivation and cellular stress. It regulates multiple cellular events, such as chromatin remodeling and cell division [142,143,144,145]. 

MCM2–7 proteins (particularly present in the chromatin-bound fraction) are all substrates of *O*-GlcNAcylation in human cells [146,147,148]. We explored the YinOYang1.2 server and found some predicted MCM *O*-GlcNAcylation sites (Table 6) [149]. Moreover, OGT directly interacts with MCM3, MCM6, and MCM7; OGT depletion decreases the interactions between MCM subunits, which subsequently impairs chromatin loading with no impact on replication rate [148]. This finding indicates that *O*-GlcNAcylation likely predominantly occurs on the dormant MCM complex. Given that OGT can be recruited to DNA damage sites [150] and that the dormant MCM complex is required to maintain genome stability [16], MCM *O*-GlcNAcylation may help ensure a rapid response to DNA damage and replication stress [148].

### 5.6. MCM Acetylation

**Figure 6:**
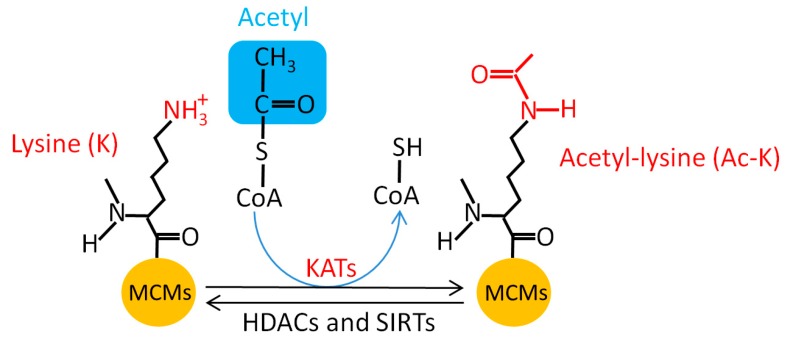
MCM acetylation. KATs, lysine acetyltransferases; HDACs, histone deacetylases; SIRTs, nicotinamide adenine dinucleotide (NAD)-dependent protein deacetylase sirtuins.

Protein acetylation covalently introduces an acetyl functional group into a substrate (Figure 6) and modulates varieties of cellular activities through acetylation of histones and non-histone proteins [151]. Several studies revealed that MCM proteins are substrates for acetylation (Table 7) [152,153,154]. Takei et al. identified MCM3-associated protein (MCM3AP) as an acetyltransferase for chromatin-bound MCM3 acetylation that inhibits the initiation of DNA replication [155]. Fatoba et al. found that the p300 histone acetyltransferase (HAT) acetylates MCM10 in both the DNA binding domain internal domain (ID) and the C-terminal domain (CTD) [156], but its acetylation elicits different biological functions in vivo; acetylation in the ID promotes MCM10 DNA binding, while acetylation in the CTD suppresses DNA binding. Meanwhile, some of the acetylation sites (Table 7) can be directly deacetylated by the nicotinamide adenine dinucleotide (NAD)-dependent protein deacetylase sirtuin-1 (SIRT1) [156]. SIRT1 depletion promotes MCM10 chromatin loading and retention during S phase and increases the replication speed. Simultaneous depletion of SIRT1 and MCM10 slows down the replication speed and fired origin distance, which suggests that MCM10 and SIRT1 synergistically modulate DNA replication progress [156]. These findings suggest that MCM protein acetylation has an important role in replication. 

### 5.7. Other PTMs of the MCM Proteins

Merbl et al. identified MCM10 as a substrate of other ubiquitin-like modifiers, such as neural precursor cell-expressed developmentally downregulated 8 (NEDD8) and interferon-stimulated gene 15 (ISG15) [129]. The biological functions of these modifications to MCM10 are unknown.

## 6. Conclusions

In sum, MCM subunits are modified by various PTMs during DNA replication under unperturbed conditions and in response to replication stress and DNA damage. Here, we summarize the functional modification sites (Table 8). Among the MCM PTMs, phosphorylation is an important replication initiation event but is also needed when replication is progressed. Most of the MCM phosphorylation sites are well understood in yeast and other model systems, but less so in human cells. Ubiquitination is also a common PTM, but its function is poorly investigated in human cells. The replication termination model mediated by MCM7 ubiquitination is, however, well delineated in yeast and *Xenopus* eggs. The biological function of other PTMs and cross-talk among different PTMs of MCM complex is largely unknown and warrants further investigation. Finally, MCMs are tightly involved in different cancers due to various gene alterations, and some MCM subunits already serve as diagnostic biomarkers. It will take time to fully understand the biological functions of the modified MCM sites that have been found thus far; however, once understood, these may open up new opportunities to develop small molecules for cancer therapy.

## Figures and Tables

**Table 1:** Human diseases associated with mini-chromosome maintenance (MCM) variants.

MCMs	Disease	Pathogenic Mutation	Reference
MCM2	Non-syndromic sensorineural hearing loss	Arg44 to Cys (R44C)	[27]
MCM4	Natural killer cell and glucocorticoid deficiency with DNA repair defect	Loss of the N-terminal sequence	[31,32]
MCM5	Meier–Gorlin Syndrome 8	2-bp deletion in exon 7; Thr466 to Ile (T466I)	[35]
MCM8	Premature ovarian failure 10	Pro149 to Arg (P149R); c.1954-1G–A splice site mutation in intron 14; c.1469–1470insTA	[38,40]
MCM9	Ovarian dysgenesis 4	c.1732 + 2T–C transition in intron 9; Arg132 to Ter (R132X); Glu495 to Ter (E495X)	[37,39]

**Table 2:** Alterations in MCM genes in human cancers.

Cancer Type	No. of Cases	MCM2	MCM3	MCM4	MCM6	MCM7	MCM8	MCM9	MCM10
Stomach adenocarcinoma	1131	3.45% (M)	-	3.71% (M)	-	5.13% (A)	4.42% (M)	-	-
Serous ovarian cancer	1191	3.78% (A)	-	3.61% (A)	-	4.11% (A)	5.04% (A)	-	4.79% (A)
Head and neck squamous cell carcinoma	1332	3.38% (A)	-	5.11% (A)	-	5.03% (A)	-	-	-
Cutaneous melanoma	892	3.03% (M)	-	-	-	-	3.03% (M)	-	3.14% (M)
Uterine endometrioid carcinoma	1242	3.22% (M)	-	3.7% (M)	-	-	-	-	-
Esophageal adenocarcinoma	255	-	6.67% (A)	-	-	12.55% (A)	3.92% (A)	3.53% (M) 3.92% (A)	-
Hepatocellular carcinoma	798	-	-	5.26% (A)	-	-	-	-	-
Invasive breast carcinoma	954	-	-	4.61% (A)	-	-	-	-	-
Pancreatic adenocarcinoma	360	-	-	3.33% (A)	-	3.33% (A)	-	-	-
Prostate adenocarcinoma	1803	-	-		3.61% (D)	-	-	6.16% (D)	-

Note: Data were obtained from www.cbioportal.org in April 2019. Alterations with a frequency >3% and patient cohort size >250 were included. A, amplification; D, deletion; M, mutation.

**Table 3:** MCM phosphorylation sites in human cancer cells.

Protein	Kinase	Phosphorylation Sites	Biological Significance	References
MCM2	CDC7	S4, S5, S7, S13, S26, S27, S31, S40, S41, S53, S108, S139, S220		[63,69,70,72,79]
		S4, S5, S7	Promotes MCM2 chromatin loading	[63]
	CDK7	S4, S5, S7, S13, S27, S40, S41, S53, T59, S108, S139		[68,69]
	CDK2	S13, S27, S40, S41, S53, S108, S139		[58,69]
	ATR	S108	Response to DNA damage	[71,73]
	CSNK2A1	S13, S27, S40, S41, S53, S108, S139		[69]
	AURKA	S220		[70,80]
	Unknown	T25, S26, T35, T39, Y137, S381, S484, S566, S754		[73,81,82,83,84,85]
		S26, S27, S40, S41, S108, S139, S170	Response to DNA damage	[78]
MCM3	CDK1	T722	Promotes MCM3 chromatin loading	[60]
		S112	Promotes MCM2–7 complex formation and chromatin loading	[59]
		S611, S719		[59]
	CDK2	S711		[58]
	ATM	S535, S728	Response to DNA damage	[71,86]
	ATR	S535	Response to DNA damage	[71]
	CHK1	S205	Negatively regulates DNA replication	[74]
	DAPk1	S160		[77]
	Unknown	S672, S711, T713, S728	Response to DNA damage	[78]
		S681, S728, S734	Response to DNA damage	[73]
		T674, T725		[73,82,83,85,87,88,89]
MCM4	CDK1	T7, T19, S32, S88, T110		[64,81,82,85,90,91,92,93]
	CDK2	T7, T19, S32, T110	Release MCM complex from chromatin	[65]
		S3, T53, S54		[64,81,82,85,90,91,92,93]
	Unknown	S31, S120, S131, S326		[81,82,84,87,88,94]
MCM5	CHK1	T633		[75]
	Unknown	S2, S315, S605		[81,82]
MCM6	Unknown	S13, S762	Response to DNA damage	[78]
		T791	Response to DNA damage	[73]
		S219, T259, S271, T278, T380, S385, S413, S758		[81,82,83,85,89,94,95,96,97,98]
MCM7	CDK1	S121		[62,81,82,84,85]
	CDK2	S121	Promotes MCM7 chromatin loading and proper mitotic exit in M phase	[62]
		S365		[62,81,82,84,85]
	LYN	Y600		[99]
	Unknown	S549	Response to DNA damage	[73]
		S314, S500, S678		[73,99]
MCM8	Unknown	S630		[81]
MCM9	Unknown	S762, S802, S1109, S1069		[81,82,85]
MCM10	Unknown	T85, S93,		[73,83,85]

CDC7, cell division cycle 7-related protein kinase; CDK7, Cyclin-dependent kinase 7; ATR, Ataxia telangiectasia and Rad3-related protein; CSNK2A1, casein kinase II subunit α; AURKA, Aurora kinase A; ATM, ataxia telangiectasia mutated protein; CHK1, checkpoint kinase-1; DAPk1, Death-associated protein kinase 1; LYN, Tyrosine protein kinase; S, serine; T, threonine; Y, tyrosine.

**Table 4:** MCM ubiquitination in human cancer cells.

Proteins	Potential Ubiquitination Sites
MCM2	K178, K216, K224, K384, K462 ^#^, K469, K476, K492, K505 ^#^, K529 ^#^, K538, K591, K613 ^※^,K722, K752, K837 ^#^, K863 ^#^, K868 ^#^, K896
MCM3	K35, K152, K207, K230, K248, K266, K270 *, K283 *, K293, K301, K315 ^#^, K351*, K413, K435*, K463, K579, K655 ^#,^ K656 ^#^, K732, K748 *
MCM4	K179, K216 ^#^, K220 ^※^, K381, K413, K439, K450, K455, K47, K478 ^#^, K536, K549, K578, K600, K627, K628, K746, K752, K762, K770, K814, K819^※^, K837, K858
MCM5	K141 ^※^, K220, K228, K256, K337, K351, K387, K392, K396, K407, K471, K499, K581, K583, K627, K696 ^※^
MCM6	K28, K102, K108 ^※^, K173, K197, K205 ^※^, K313, K326, K332, K365, K402, K407 ^※^, K422 ^※^, K486, K490, K517, K588, K599, K611, K643, K646, K654^※^, K733, K744, K754, K769, K775, K796
MCM7	K4, K10, K15, K28 ^※^, K29 ^※^, K32, K75 ^※^, K89, K96, K145, K159 ^#^, K174, K231 ^※^, K236, K305, K308, K335, K351, K352, K387, K471, K557 ^※^, K569, K596 ^※^, K627, K641, K648
MCM8	K651
MCM9	K296, K446
MCM10	K134, K139, K313, K493, K520, K627, K665

^#^ response to Etoposide, refers to Reference [78]; * Kelch-like erythroid-derived Cap-N-Collar homology protein (ECH)-associated protein 1(KEAP1)-dependent, refers to Reference [107]; ^※^ involved in proteasomal degradation, refers to Reference [106]; other sites refer to References [104,105]. K, lysine.

**Table 5:** MCM small ubiquitin-like modifier (SUMO)ylation sites in human cancer cells.

Proteins	Potential Sumoylation Sites
MCM3	K248, K266, K732, K736
MCM4	K439, K477
MCM7	K4, K10, K15, K32, K159, K174, K231, K236, K308
MCM10	K134, K139, K313, K362, K482, K511, K534, K627, K665, K669, K675, K682, K695, K746, K762, K769, K854, K869

Sites refer to References [141].

**Table 6:** Predicted *O*-N-acetyl-D-glucosamine (GlcNAc)ylation sites for MCMs.

Proteins	Potential O-GlcNAcylation Sites
MCM2	S5, T25 **, S26, S27, S31, T35, T308, T313, S540, T546, S566, S608, T625, T845
MCM3	S118, T154, S170, S171, S302, S348, T368, T369 *, T383, S595 *, T610 *, S781
MCM4	S2 **, S3 *, S6, T7 *, S9, T19, S26 **, T53, S54 *, S70, S71, S77, S87 *, S97 *, S105, T369, T391, S406 *, T533, T611, S703, T778
MCM5	T111, S135 *, S136, T476, S600 *, S647, S654
MCM6	T266, S399, T419, S420 *, S507, S607
MCM7	S143, S401, T404, T405, T601, T654, S670 **, T717
MCM8	S338, S361, T484, S485, S555, S574, S621, T635, T839
MCM9	S91, S170, S171, T376, S468, T564, S659, S725 **, S727, T767 *, S768, S777, T864, T871 *, S874, S878, T879, S898, T946, S952, T990 *, S1067 **, S1069 *, S1088, T1092 **, T1093 *, S1099 *, S1143
MCM10	T96, T137, S143, S150, S171, T186, T192, S196, S202 **, S203 **, S205, T208 **, T217 *, S237, S261 *, S465 *, S555, S593, S598 *, S599 **, S600, T610 *

* Represents high potential. Prediction sites data were collected from YinOYang1.2 server in April 2019 (http://www.cbs.dtu.dk/services/YinOYang/).

**Table 7:** MCM acetylation in human cancer cells.

Protein	Potential Sites
MCM2	K216, K469, K742, K896
MCM3	K152, K556, K559
MCM4	K123, K220, K439, K450, K627, K819, K858
MCM5	K220, K387, K392, K396, K581, K696
MCM6	K313, K344, K517, K599, K643, K646, K775
MCM7	K28, K29
MCM10	K267 *, K312 *^, #^, K318 *, K390 *^, #^, K657 *, K664 *, K681 *, K683 *^, #^, K745 *^, #^, K761 *^, #^, K768 *^, #^, K777, K783 *, K853 *

Sites refer to References [152,153,154,156]. K, lysine; M, methionine. * Refers to lysine residues acetylated by p300 [156]. ^#^ Refers to lysine residues deacetylated by SIRT1 [156].

**Table 8:** Summary of functional modification sites for MCMs.

Protein	Modification	Modified Sites	Biological Significance	References
MCM2	Phosphorylation	S4, S5, S7	Promotes chromatin loading	[63]
		S108	Phosphorylated by ATR upon DNA damage	[71]
		S27, S41, S139	Promotes replication; response to DNA damage	[63,68,78]
	Ubiquitination	K462, K505, K837, K863, K868	Response to DNA damage	[81]
MCM3	Phosphorylation	S112	Promotes MCM2–7 complex formation and chromatin loading	[59]
		S205	Phosphorylated by CHK1, negatively regulates DNA replication	[60]
		S535	Phosphorylated by ATM upon DNA damage	[71]
		T722	Promotes chromatin loading	[60]
		S672, S711, T713, S728	Response to DNA damage	[78]
	Ubiquitination	K315, K655, K656	Increased upon DNA damage	[81]
MCM4	Phosphorylation	T19, S32, T110	Promotes MCM complex unloading from chromatin	[65]
	Ubiquitination	K216, K478	Response to DNA damage	[81]
MCM6	Phosphorylation	S13, S762	Response to DNA damage	[78]
MCM7	Phosphorylation	S121	Promotes chromatin loading and proper mitotic exit	[62]
	Ubiquitination	K159	Response to DNA damage	[78]
